# Implementation Barriers and Facilitators of an Integrated Care Initiative Targeting Socioeconomically Vulnerable Groups

**DOI:** 10.5334/ijic.10182

**Published:** 2026-08-18

**Authors:** Jonne G. ter Braake, Annefrans F. T. M. van Ede, Tara Kwakkernaat, Maud J. Verhoeven, Jessica C. Kiefte-de Jong, Rimke C. Vos

**Affiliations:** 1 Health Campus The Hague/Department of Public Health and Primary Care, Leiden University Medical Center, The Hague, The Netherlands

**Keywords:** public health, integrated care, community health services, social determinants of health, health equity, prediabetic state

## Abstract

**Introduction:**

The incorporation of integrated care initiatives for socioeconomically vulnerable groups into standard care is rare. Insight into implementation barriers and facilitators to select strategies for further implementation and upscaling are therefore needed.

**Description:**

This study evaluated the implementation of an integrated care initiative adopting interprofessional collaborative practice of patient-centred care for citizens with prediabetes in the form of group consultations.

**Results:**

Key barriers included insufficient involvement of citizens in the design of the group consultations, which could be overcome by involving patients/consumers and family members in the design of the group consultations. Additionally, we identified a lack of embedding in the current healthcare and social support systems and fragmented financing systems as barriers. By developing a formal implementation blueprint, obtaining formal commitments, using other payment schemes, and developing resource sharing agreements, these barriers could be addressed. Finally, inadequate evaluation was identified as a barrier. This could be improved by capturing and sharing local knowledge and developing and organising a quality monitoring system. Key facilitators were the widespread enthusiasm for the concept of group consultations, a sense of urgency for change, and the adaptability of the initiative to the local context.

**Conclusion:**

Meaningful citizen involvement in the design of an integrated care initiative, such as group consultations, is a key determinant of implementation when targeting socioeconomically vulnerable groups. Although there is broad support for interprofessional collaborative practice, the organisational and governance structures required for their sustainable implementation remain underdeveloped.

## Background

The prevalence of prediabetes was found to be higher than previously estimated in the Netherlands, with approximately 21% of males and 15% of females between 40 and 75 years of age having prediabetes [1]. Prediabetes is characterised by elevated blood glucose levels that are not yet high enough to be classified as diabetes [2]. Around 70% of people with prediabetes develop type 2 diabetes within ten years [2,3]. Both the onset of prediabetes and the deterioration from prediabetes to diabetes were found to be socially patterned, with socioeconomically vulnerable people having an increased risk of developing (pre)diabetes compared to more affluent peers [1,4]. To prevent the deterioration from prediabetes to diabetes, lifestyle interventions have proven effective in restoring normal blood glucose levels and improving cardiometabolic markers [2,5,6]. However, social-contextual constraints can hinder lifestyle changes if not properly addressed, for example individual characteristics including socioeconomic position and neighbourhood characteristics [7,8]. This can place socioeconomically vulnerable people at a further disadvantage. Although initiatives are available to mitigate these social constraints, many citizens do not receive appropriate support matched to their needs because of fragmentation of healthcare and social support (unpublished findings).

Across different contexts, there is growing recognition of the need to integrate social support and medical care. Experts emphasise the potential of integrated care to deliver personalised care, enhance health outcomes, and foster greater equity within the healthcare system [7,9,10]. Using an integrated care approach, underlying issues hindering lifestyle change, such as debts or unemployment, could be addressed first before the focus is shifted to stimulating lifestyle change [7]. This approach has the potential to improve health especially in socioeconomically vulnerable people [10].

Despite the promise of integrated care initiatives, the incorporation of these initiatives into standard care is rare [11,12,13]. Previously identified barriers include difficulties in sharing information between professionals and/or organisations [12,14,15,16,17], funding [12,13,14,15,16,17,18], and the accessibility of locations [15,16]. Facilitators include effective communication and collaboration between professionals [12,14,15,16,17,19]. Facilitators identified when specifically targeting socioeconomically vulnerable groups include citizen engagement and needs assessment and the use of a positive health approach [18]. From a positive health perspective, health encompasses more than just addressing disease and illness; it also involves an individual’s perceived control and capacity to manage life’s challenges [20]. Overall, information remains scarce on whether the identified barriers and facilitators in the implementation of integrated care initiatives are similar when specifically targeting socioeconomically vulnerable groups, despite being especially beneficial for these groups [15,21,22,23].

We therefore explored implementation determinants in this specific context. For this, we evaluated implementation of group consultations that bridge the medical and social domain, targeting citizens with prediabetes living in socioeconomically vulnerable neighbourhoods in The Hague, the Netherlands, as a case example. These group consultations are characterised by interprofessional collaborative practice of patient-centred care for individuals with prediabetes and were initially provided in two neighbourhoods in The Hague, The Netherlands. An implementation team of various professionals is now exploring upscaling to additional neighbourhoods. For this purpose, insights into implementation barriers and facilitators are needed, as well as the selection of context-sensitive strategies for implementation and upscaling. Therefore, we aim to evaluate the implementation barriers and facilitators of this integrated care initiative which targets socioeconomically vulnerable groups and select strategies to improve implementation and upscaling.

## Methods

### Study design and setting

We used a qualitative design combining individual interviews and a focus group to evaluate barriers and facilitators of the implementation of an integrated care initiative that adopts interprofessional collaborative practice of patient-centred care for individuals with prediabetes in the form of group consultations [24].

The group consultations were developed by a primary care physician motivated by her observation that socioeconomically vulnerable people with prediabetes frequently returned to her practice with recurring physical complaints. These complaints often stemmed from unresolved underlying social constraints that remain unaddressed in traditional primary health care. She decided to adopt a Population Health Management approach [25] and use interprofessional collaborative practice to better adjust care to the multifaceted needs of this vulnerable group. She collaborated with a neighbourhood sports coach, a social worker, and medical staff to design a group consultation, aiming to familiarise citizens with available local support tailored to their needs. During a single 90-minute session, various professionals from different disciplines, including but not limited to a primary care physician, neighbourhood sports coach, and social worker, provide a group consultation where the interrelatedness of social and medical problems is discussed from a positive health perspective [20], a physical check-up is conducted, and a warm handoff is arranged for initiatives in the neighbourhood based on the social and medical needs of the participants. Follow-up trajectories could include sessions with the neighbourhood sports coach, a lifestyle coach, or social worker. A group follow-up session is scheduled six months after the initial session. The groups generally consisted of between four and 15 participants. These group consultations were initiated in two vulnerable neighbourhoods in 2020. The neighbourhoods are assigned a higher deprivation score than average in The Hague, based on the wealth, educational level and employment (SES-WOA score) in the neighbourhood [26]. In 2023 scaling commenced to additional neighbourhoods in The Hague. An implementation team, including health care professionals, community representatives, and researchers, has been involved in the upscaling of the intervention.

### Theoretical frameworks and data collection

**Figure 1:**
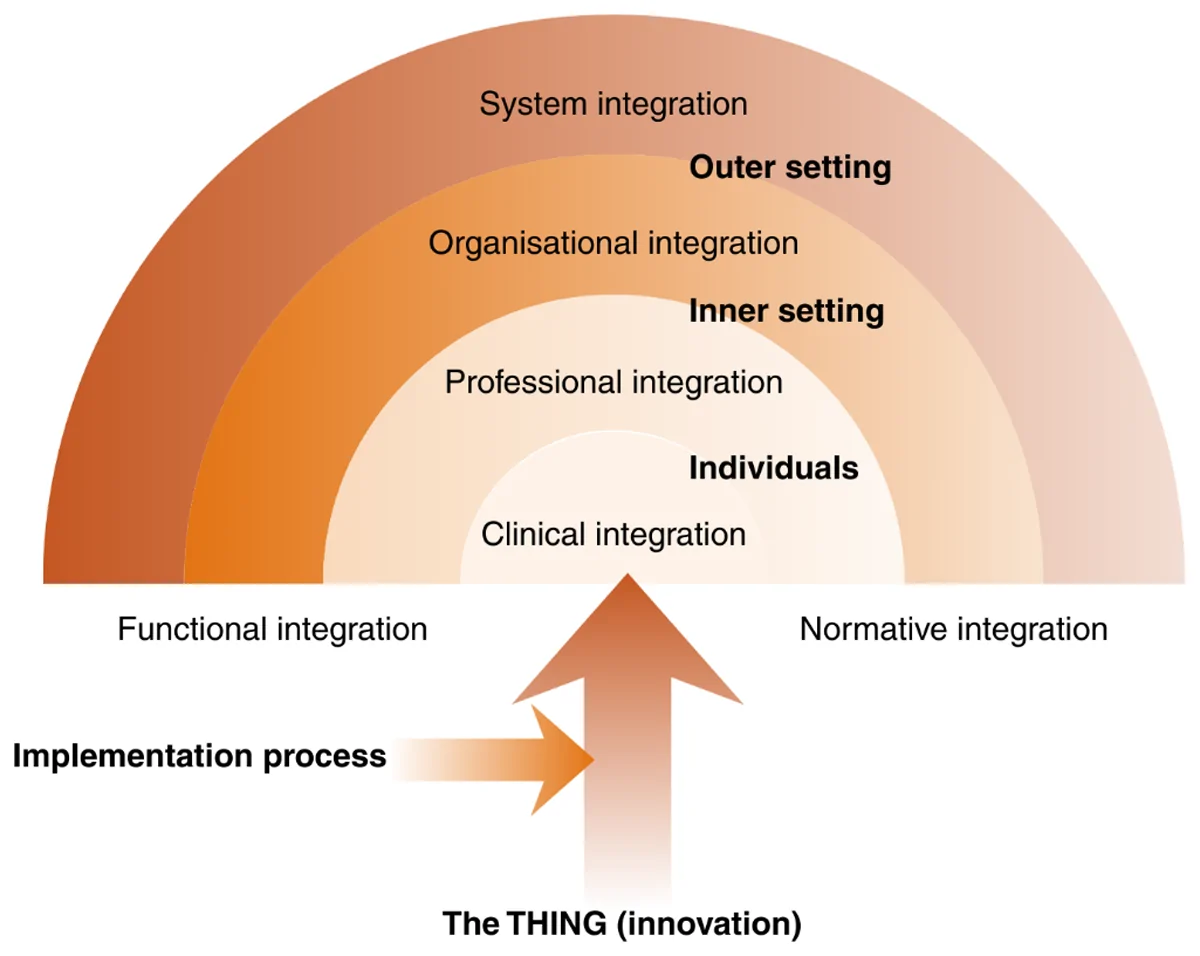
The Rainbow Model of Integrated Care and Consolidated Framework of Implementation Research. Adapted from: Valentijn et al. (2013) [28] and The Center for Implementation (2025) [29].

We used the implementation determinant framework Consolidated Framework of Implementation Research (CFIR) in combination with the Rainbow Model of Integrated Care (RMIC) to guide data collection and analysis (Figure 1).

The CFIR offers a standardised structure for identifying barriers and facilitators of implementation, enabling knowledge generation on what works where, beyond the specific research settings [27]. This framework identifies six domains: innovation, the outer setting, the inner setting, individuals, and the implementation process; each domain is composed of a number of constructs [27].

The RMIC was developed to better understand the concept of integrated care [28]. The model conceptualises the process of integrating care through six dimensions: clinical, professional, organisational, system, functional, and normative integration [28]. To deliver integrated care, integration across all levels is needed. The first four dimensions explore integration from the micro to the macro level. *Clinical integration* is defined as the coordination of patient care activities and whether services improve an individual’s overall well-being; *professional integration* explores the collaboration among healthcare professionals across disciplines; *organisational integration* analyses the alignment and cooperation among healthcare organisations; and *system integration* considers the broader health system’s policies, regulations, and structures that influence care delivery [28]. *Functional integration* supports and links integration over these four levels, and *normative integration* is defined as the development and maintenance of a shared mission, vision, values and culture over the four levels [28].

We employed the CFIR’s Expert Recommendation for Implementing Change (ERIC) framework for the selection of implementation strategies matched to the identified barriers and facilitators [30]. This framework defines implementation strategies based on input from a wide range of stakeholders [30]. By using this framework, we ensured the unambiguous use of previously defined strategies.

Data collection took place between April and October 2024 and is described in detail below.

### Interviews

Semi-structured interviews guided by the CFIR framework were conducted with a varied group of stakeholders directly or indirectly involved in the organisation of the group consultations. The interviewees included but were not limited to people with lived experience, community leaders, medical and social care professionals, members of the expert team organising the group consultations and representatives of the municipality, health insurance and national government. The interviews were conducted in-person, via videocall or by telephone, depending on the preference of the interviewee. We used purposive sampling guided by the RMIC levels to select participants with knowledge and perspectives on varied aspects of implementation and integration. Professional networks of implementation team members were used in this process. A priori the sample size to reach meaning saturation was estimated at approximately 26 interviews, as we expected partial overlap in barriers and facilitators over the four levels of the RMIC. Meaning saturation was reached when no new barriers or facilitators were identified nor refinement was needed [31]. As CFIR includes a comprehensive list of constructs, a selection of topics was chosen prior to each interview based on the knowledge and expertise of the respondent. Additional constructs were discussed when deemed appropriate.

The majority of interviews were conducted by JGB, six together with a student (TK or MV) and three by a student alone (TK or MV). The interviews were recorded and transcribed verbatim.

### Focus group

A semi-structured in-person focus group was organised with a women’s group volunteering in one of the neighbourhoods. The group consisted of 16 women, 11 of whom had previously participated in a group consultation. The focus group provided further insights into barriers and facilitators at the clinical level of implementation and was used to validate and enrich findings from the interviews from a citizen perspective. The focus group was not recorded. Instead, field notes were made during the session and subsequently expanded by two authors (RCV and JGB) who were present during the discussion. This approach was found to be more appropriate because audio recording potentially changes the group dynamic as participants experience nervousness over their voices being recorded [32].

### Data analysis

We used Framework analysis, a descriptive qualitative approach originating from policy research, to analyse the data obtained during interviews and the focus group [33]. Framework analysis is a method to describe and interpret what is happening in a particular setting via five iterative steps: familiarization, identification of a thematic framework, indexing, charting, and mapping and interpretation [33]. All steps were conducted by the first author (JGB) in collaboration with a ‘critical friend’ [34], AFTME, and overseen by RCV. The five steps were discussed and revised during critical dialogue over several sessions. The dialogue provided a theoretical sounding board where the thought process of the first author was evaluated, alternative interpretations were coined, and interpretations were revised accordingly.

A priori, the CFIR was identified as a suitable framework to guide data analysis. Based on the familiarisation phase, deductive use of the CFIR domains and constructs for indexing was deemed appropriate. To enable further discussion of the indexing in a time-efficient manner, a step between indexing and charting was added. We first arranged the indexed data according to the CFIR constructs via a ‘cut and paste’ approach, after which adjustments to the indexing were made to reach consensus. A distilled summary per construct was then charted, ordered by the RMIC levels. We used mapping and interpretation to define barriers and facilitators per construct and RMIC level using the distilled summaries. This process was conducted iteratively until meaning saturation was reached.

Then, related barriers and facilitators were combined by JGB and RCV and a selection was made to identify those most prominently needing strategies to overcome the barrier or leverage the facilitator. This process was evaluated by two members of the implementation team and an independent expert familiar with the intervention who were not involved in this study.

We matched the implementation strategies to the selected barriers using CFIR’s ERIC matching tool [35].

### Patient and public involvement

Throughout the development and upscaling of the group consultations, people with lived experience and community leaders were involved. These people with lived experience are members of the community of the neighbourhoods where the group consultations took place. As included in the sampling description, some people with lived experience and community leaders were included in this study as participant in the interviews. Also, a focus group was conducted in the community with a focus on the implementation of the group consultations and a reflection on their role in this process. People with lived experience were not directly involved in the design, conduct, or reporting of the academic work that is presented in this manuscript.

### Ethics approval and consent to participate

The Medical Ethical Review Board of Leiden University Medical Center mandated the review of research not falling under the Dutch Medical Research with Human Subjects Law (nWMO) to individual nWMO committees at Leiden University Medical Center. The nWMO committee reviewed the proposal and provided a declaration of no objection (non-WMO approval number: 24–3066). This study has been further approved by the scientific committee of the department of Public Health and Primary Care at Leiden University Medical Center (Reference number: WSC-2024–37).

All the interviewees provided written informed consent using an informed consent form approved by our institution. The participants in the focus group gave verbal consent after being informed about what data we would gather and how. No personal data were gathered during the focus group.

## Results

We identified 50 unique barriers and 30 unique facilitators of the implementation of the group consultations (Additional file 1) based on 28 interviews and one focus group (n = 16). Ten more determinants were identified; these determinants were present in some neighbourhoods and absent in others and functioned as facilitators when present and barriers when absent. From these 90 determinants, we selected five main barriers and three main facilitators (Table 1). Implementation strategies were identified for the main barriers. The main barriers, facilitators, and strategies are further discussed below.

**Table 1:** Selected barriers and facilitators, their CFIR determinant and level of RMIC, with illustrative quotes.


	DETERMINANT	DETERMINANT CFIR	LEVEL OF THE RMIC	QUOTES

**Barriers**	Insufficient involvement of citizens in the design of the group consultations. Important sub barriers: – The examples used during the group consultations are not tailored to the cultural background of participants. – Detachment between residents and professionals reduces trust – Many citizens think it is important to have separate groups for men and for women	Process – Assessing needs Innovation – Innovation design Outer setting – Local conditions Outer setting – Local attitudes	Clinical/normative integration	Q1: ‘But it also has to do with: you put things in front of me that I don’t like. You want me to cook certain things that I don’t know how to cook.’ – Clinical level Q2: ‘This is a theme about losing weight. Especially among women, it is a private theme. It’s a… It also has to do with self-confidence, self-confidence. So in a group where there are men there, you’re not really going to be able to get that out, so out of, that a woman is going to tell something so intimate.’ – Clinical level Q3: ‘I also hope that the consultations will be carried even more by the local residents themselves, so that it really comes from the community. And that they also get a voice in designing the consultations, for example.’ – Professional level Q4: ‘Adjusting [the intervention] to the group, there’s still a lot to be done in that, in my opinion’ – Organisational level

	Gap between the group consultations and local follow-up initiatives	Innovation – Innovation design	Professional/functional integration	Q5: ‘At this one there were lots of dieticians and someone from, for people with financial problems, for people with financial, you name it. All sorts of things were there. Eating problems.[…] Heart foundation was also there and I understood that all. Also understand why they were invited. I was only wondering like: what’s the added value? The performance did go nicely. It was nice. The atmosphere and everything. That was all right. But after that I did notice some people were like: what am I here for? What is the actual help?’ – Clinical level Q6: ‘Often they are in different trajectories and then it may occur that I lose track of them, but they are still actually working on a trajectory, which also originated from the group consultation. So that link, if indeed something is documented or streamlined, that we can also better keep track on that. […] So often you lose the people who try something and it doesn’t work out and then think: it didn’t work out, I’ll stop. Whereas a moment of contact or a signal of: this person has stopped, if we can pick this up again, it will be easier to pick up the trajectory again.’ – Professional level

	The lack of embedding of the group consultation into formal agreements/structures. Important sub barrier: Roles within the implementation team are unclear.	Process – Planning Individuals – Implementation leads	Organisational/functional integration	Q7: ‘For that to spread out completely in the neighbourhoods, there is – I think – still a lot depending on enthusiastic colleagues – professionals – rolling that out. Ideally, you would like to secure that in a function within…. In a neighbourhood, for instance. That it is not person-dependent, but more embedded.’ – Organisational level

	Fragmented financing systems	Outer setting – Local conditions; Inner setting – Available resources	System/functional integration	Q8: ‘[…]funding is a problem and has been for some time, because on the one hand you have the angle from medical support and support from the social domain, with the health insurer and municipality looking at each other like: who is going to pay for what?’ – Organisational level Q9: ‘And yes, you know, I think this is actually another one of those typical annoying cases of the responsibility lies everywhere and therefore nowhere.’ – System level

	Inadequate evaluation of effectiveness and the implementation process.	Process – Reflecting and evaluating	Organisational/functional integration	Q10: ‘Sure she asked that at some point. Sure that that also came up and I may have mentioned something. But not very well-founded and not very well thought out, I think.’ – Professional level Q11: ‘But to put it very simply: to get a payment title for group consultations, you will have to present results from the field, if you would want to pay for it from the ZVW [Health Insurance Act].’ – System level

**Facilitators**	Widespread enthusiasm for the concept of the group consultations	Innovation – Innovation relative advantage Inner setting – Mission alignment	Professional/normative integration	Q12: ‘During group consultations, we notice that people truly feel heard.’ – Professional level Q13: ‘Well, if you go to one of those group consultations […] and you find out there: who is that dietician? Oh, […] that seems like a really nice person. And you can make an appointment right away, that’s of course perfect. Then you really have, just that warm referral.’ – Organisational level

	Sense of urgency for change among stakeholders	Inner setting – Tension for change	Professional/normative integration	Q14: ‘I said: well, I’m open to that kind of thing too, just offering care in a different way instead of just one-on-one. Because sometimes you think: “Hello, does it sink in?” or “what about the advice I give?” And then when someone says, for example, after ten times, “I’ve never heard that before.” Then I think, I think I’ve already told them ten times.’ – Professional level

	Adaptability of the initiative to the local context	Innovation – Innovation adaptability	Clinical/normative integration	Q15: ‘Yes, because you see that that’s important for success. That you respond to the needs in the neighbourhood’ – Professional level Q16: ‘If there are a lot of questions regarding quitting smoking, then we’ll ask aa smoking cessation coach to join. So that way you can adjust it a little bit each time too.’ – Professional level


### Barriers

The first main barrier was the insufficient involvement of citizens in the design of the group consultations, which was identified as being part of the ‘*assessing needs*’ determinant of the ‘*process*’ domain of CFIR. This barrier was mapped on the clinical level of the RMIC under normative integration. The limited involvement of citizens in the design and implementation process resulted in several sub barriers, such as inadequate tailoring of the intervention to the cultural background of participants and reduced trust (Table 1 Quote (Q) 1–4).

Secondly, respondents reported that a gap remained between the group consultations and local follow-up initiatives, such as sessions with the neighbourhood sports coach, a lifestyle coach, or social worker. This barrier was categorised under ‘*innovation design*’, part of the ‘*innovation*’ domain and influenced functional integration on the professional level. Two distinct gaps were reported. The participants felt that they did not receive adequate information about the follow-up steps, thereby preventing the initiation of a follow-up trajectory (Table 1 Q5). The professionals mentioned that when they lost touch with participants, they were unaware of whether these participants were still seen by other colleagues and thus “in the system” or fell off the professionals’ radar. Due to the lack of a structured feedback system, professionals felt demotivated to keep track of lost participants (Table 1 Q6).

The third barrier we identified was the lack of embedding of the group consultation into formal agreements/structures. This barrier influenced functional integration on the organisational level was categorised as a determinant of ‘*planning*’ under the ‘*process*’ domain. Currently, the initiative relies on efforts from various enthusiastic professionals and partners that support continuation, as described later under facilitators. However, participants mentioned the importance of embedding the initiative into formal structures to safeguard sustainability and scaling regardless of enthusiastic professionals potentially switching positions or not being able to put in the same effort (Table 1 Q7).

Guaranteeing the sustainability of the initiative related to the fourth barrier: fragmented financing systems. This determinant was identified as functional integration on the system level and categorised under both the ‘*local conditions*’ in the ‘*outer setting*’ and ‘*available resources*’ in the ‘*inner setting*’. The respondents elaborated on the complexity of the Dutch health system, where healthcare and social support are regulated and financed according to two different laws with different paying parties, and which lacks formal responsibility for health and prevention. Interviewees explained that stakeholders (partly) responsible for financing this initiative point fingers to each other to take initiative (Table 1 Q8 and Q9). No sustainable payment agreements have been made yet, thereby causing the initiative to rely on temporary funding, which threatens sustainability.

The last main barrier was inadequate evaluation of effectiveness and the implementation process. This barrier was also identified as functional integration on the organisational level under the ‘*reflecting and evaluating*’ construct of the ‘*process*’ domain. This barrier was multifaceted. The interviewed professionals had different perceptions of whether the process is currently being monitored. While some described some sort of process evaluation and monitoring system, others mentioned not being included in this process (Table 1 Q10). Overall, there seemed to be no clear structure in the process or effectiveness evaluation. The respondents also emphasised the difficulties associated with evaluating the effectiveness of preventative initiatives. They explained that not all participants change their behaviour right away; some might forget about it for some time and then after an additional trigger use the information received. Long-term evaluation is needed to capture these delayed effects. The respondents at the system level emphasised the importance of evaluating the effectiveness to secure sustainable funding (Table 1 Q11).

### Facilitators

The first main facilitator we identified was the widespread enthusiasm for the concept of group consultations among involved stakeholders. We categorised this facilitator of normative integration on the professional level under ‘*innovation relative advantage*’ in the ‘*innovation*’ domain and ‘*mission alignment*’ in the ‘*inner setting*’. Professionals were especially positive about the peer support resulting from the group setting (Table 1 Q12) and that the physical check-up handed participants tangible tools for change. Having various professionals present and arranging a warm handoff for follow-up care was also seen as an important benefit of the group consultations (Table 1 Q13).

Additionally, a sense of urgency for change was also found to facilitate normative integration at the professional level and categorised under ‘*tension for change*’ in the ‘*inner setting*’. Professionals expressed being fed up with the current system because citizens frequently returned to their practice with recurring physical complaints and questions (Table 1 Q14). This motivated them to experiment with alternative ways of working, such as group consultations.

Finally, we identified the adaptability of the initiative to the local context as a facilitator of normative integration at the clinical level, under the ‘*innovation adaptability*’ of the ‘*innovation*’ domain. The respondents underlined the importance of adapting these types of initiatives to the local context where they are implemented (Table 1 Q15), for example, considering cultural sensitivity. They emphasised that these groups consultations allow adaptation to various local contexts due to the flexible character of the different components, for example, by focusing on specific themes that are relevant in neighbourhood (Table 1 Q16).

### Strategies

We identified eight strategies that could be employed to improve the implementation of the group consultations (Table 2). We selected the strategy ‘*involve patients/consumers and family members*’ to improve the involvement of citizens in the design of the group. Methods to employ this strategy could include training health champions, people with lived experience who are motivated to help others, to aid in the recruitment of participants, preparation of group consultations, be present during group consultation, and guide participants after the initial group consultation. They can represent the local community and ensure that the intervention is adjusted to the needs of the participants.

**Table 2:** Main identified barriers and the matched strategy.


BARRIER	IMPLEMENTATION STRATEGY FROM ERIC

Limited involvement of citizens in the design of the group consultations.	Involve patients/consumers and family members

Gap between the group consultations and local follow-up initiatives	Organise clinician implementation team meetings Involve patients/consumers and family members

The lack of embedding of the group consultation into formal agreements/structures, resulting in dependence on the intrinsic motivation of involved partners for continuation of the initiative.	Develop a formal implementation blueprint Obtain formal commitments

Fragmented financing systems	Use other payment schemes Develop resource sharing agreements

Inadequate evaluation of effectiveness and the implementation process.	Capture and share local knowledge Develop and organise quality monitoring systems


We identified the strategies ‘*involve patients/consumers and family members*’ and ‘*organise clinician implementation team meetings*’ to bridge the gap between the group consultations and local follow-up initiatives. The aforementioned health champions could guide participants during and after the initial group consultation to find appropriate support or care. Additionally, clinician, or in the case of a cross-domain initiative, interdisciplinary, implementation team meetings could be employed to develop a feedback system between professionals after the initial group consultation to increase involvement. Interdisciplinary team meetings with involved professionals could be organised to monitor progress and discuss how to handle loss to follow-up among participants. To support this process, the use of ICT tools could be explored to aid communication between professionals.

To embed the group consultations into formal agreements/structures, two strategies were selected: ‘*develop a formal implementation blueprint*’ and ‘*obtain formal commitments*’. By creating a roadmap for implementation, the fixed and flexible parts of the group consultation can be determined. Roles and procedures could then be formalised and embedded via formal agreements.

To ensure sustainability, ‘*use other payment schemes*’ and ‘*develop resource sharing agreements*’ were selected to overcome fragmented financing systems. Local agreements with the municipality and health insurance companies or national agreements are required for the payment structure.

Finally, we selected ‘*capture and share local knowledge*’ and ‘*develop and organise quality monitoring systems*’ to promote systematic evaluation of the effectiveness of the group consultations and their implementation process. Iterative quantitative and qualitative evaluations would promote implementation, including the formulation of results and outcomes, a structured data gathering process, and a formal process evaluation.

## Discussion

We evaluated the implementation barriers and facilitators of an integrated care initiative that adopts interprofessional collaborative practice of patient-centred care for individuals with prediabetes in the form of group consultations, specifically targeting socioeconomically vulnerable groups in the Hague, the Netherlands. The use of CFIR in combination with RMIC enabled the generation of knowledge about what works in different contexts, extending beyond the specific research setting. Based on these findings, we propose strategies to mitigate key barriers to improve implementation and upscaling.

A key barrier was insufficient citizen involvement in the design of the intervention, a finding that aligns with previous research on the implementation of integrated care initiatives targeting socioeconomically vulnerable groups [18]. This threatened normative integration – defined as the development and maintenance of a shared mission, vision, values and culture over the clinical, professional, organisational and system levels of the RMIC [28] – on the clinical level, as the content of the intervention was not adequately tailored to the participants’ needs. This is noteworthy given that people with lived experience and community leaders were involved from an early phase, underscoring the inherent complexity of meaningful citizen involvement when properly adjusting interventions to this target group. This complexity is shaped by several interacting factors. A fundamental source of tension is the diverging ways in which professionals and socioeconomically vulnerable citizens conceptualise health: whereas professionals tend to emphasise well-being, citizens from vulnerable neighbourhoods more often perceive health as the absence of disease [36]. Such conceptual misalignment can be further compounded by professional dominance in practice – professionals have been found to take over from citizens, thereby discouraging further participation [37]. Moreover, citizen participation may prove counterproductive when decision-making authority remains concentrated among professionals [38]. These dynamics suggest that simply involving patients/consumers and family members in the design process and local adaptation of the intervention, as recommended by the ERIC matching tool, may not be sufficient. Deliberate and methodologically informed approaches for citizen involvement are required for improved normative integration.

Since every local context is unique, citizen involvement would remain important when scaling the intervention to new neighbourhoods or cities. This process is facilitated by the adaptability of the intervention, as the intervention is suitable for adaptation based on local needs while keeping core elements the same.

The strategy of involving patients/consumers and family members was also proposed to bridge the gap between the group consultations and local follow-up initiatives, ensuring that participants receive appropriate and context sensitive information to choose suitable follow-ups. Additionally, organising interdisciplinary team meetings was suggested to establish a feedback system, thereby improving functional integration; so the supporting of and linking integration between the four levels of the RMIC [28]. This way, follow-up and lost-to-follow-up can be monitored and discussed on a regular basis.

Two key facilitators of implementation were the sense of urgency for change and the widespread enthusiasm for the concept, which played a critical role in fostering normative integration. The perceived urgency for change and mission-driven motivation among initiators have previously been reported to drive bottom-up initiatives [39]. However, these other initiatives did encounter resistance from professionals who were supposed to collaborate in the new initiative, as they perceived the novel way of working as a violation of their professional autonomy [39]. We potentially did not pick up on this phenomenon in our study because we only included early adopters, so professionals who were enthusiastic about the intervention and therefore decided themselves to participate [40]. It would be valuable to reevaluate these determinants of implementation during the process of adoption into standard care.

The enthusiasm of professionals driving implementation might also have had an unintended side effect: the strong motivation of professionals to go the extra mile could have reduced the perceived necessity of embedding the intervention into formal health and social care structures. The fragmented financing systems further complicated functional integration, in line with previous findings in both the Dutch context [13,18,39,41,42] and internationally [12,14,15,16]. So although normative integration from the professionals’ perspective seemed to be going well, functional integration emerged as a challenge. This is a common phenomenon in bottom-up initiatives, where factors such as funding do not necessarily impede the initiation of change but become a barrier when initiatives move toward incorporation into standard practice [39]. For integrated care initiatives, there is an additional layer of complexity since, in the Netherlands, medical care and social support are governed by separate laws and financed through distinct budgets, thereby making the financing of these initiatives particularly challenging. This fragmentation often leads to the discontinuation of promising or even proven effective initiatives after their pilot phase due to the absence of sustainable payment agreements [13,41]. So, despite widespread recognition of the benefits of interprofessional collaborative practice, the absence of suitable governance structures including long-term payment agreement hinders their sustainable integration into practice.

The selected strategies to enhance functional integration include developing a formal implementation blueprint, obtaining formal commitments, exploring other payment schemes, and creating resource-sharing agreements. However, these last two strategies are complicated as real-world examples of sustainable payment models for integrated care initiatives that combine social and medical support remain scarce in the Dutch context. Nies et al. (2021) even described the separate reimbursement schemes for medical and social support as an ‘unsurmountable barrier’ [13]. Payers of both medical and social care – health care insurers and municipalities, respectively – are reluctant to invest in preventative activities because potential cost savings could fall under another budget, the so-called ‘wrong pocket-problem’ [41]. Therefore, in line with our findings, they tend to point fingers to each other to take initiative. This underlines the importance of creating resource-sharing agreements, including how to deal with potential savings, and indicates the need to explore (alternative) payment models within or beyond the current siloed health and social system to address such issues.

To achieve embedding into formal structures and sustainable financing, systematic evaluation of the intervention’s effectiveness and implementation process are usually a prerequisite [43]. To address this, we propose capturing and sharing local knowledge and developing a quality monitoring system to drive ongoing improvements. The development of a learning health system would be an elegant example where these two strategies are combined [44]. In a learning health system, research and health care delivery, or integrated care delivery, are fused by embedding advanced research methods within the care trajectory [44]. Existing data infrastructures and insights from participants and professionals are used together to create rapid improvement cycles [44]. To improve equity, citizens should be involved in this process and it should be monitored whether all relevant perspectives are represented or whose voices are absent [44].

### Strengths and limitations

A key strength of this study is the use of the RMIC framework in participant selection. By incorporating clinical, professional, organisational, and system-level perspectives, we ensured a diverse sample of respondents, representing various dimensions of implementation. Additionally, the use of the updated CFIR for data collection facilitated a broad focus during interviews and throughout the data collection process. During data analysis, this standardised determinants framework enabled the generation of knowledge about what works in different contexts, extending beyond the specific research setting. The combination with the RMIC facilitated interpretation of the findings in the context of integrated care. This approach carried the risk of a potential tunnel vision, focusing on the constructs within the framework and possibly overlooking themes outside its scope. However, this risk is considered minimal. The CFIR was developed by integrating earlier frameworks, including the Promoting Action on Research Implementation in Health Services (PARiHS) and the Practical, Robust Implementation and Sustainability Model (PRISM), and has since been extensively used and iteratively revised based on user feedback in implementation science, thereby ensuring a thorough and comprehensive scope (27). A limitation is the inclusion of early adopters in the study, since the intervention has not yet been adopted into standard care. As a result, clinical, professional, and organisational integration may have been portrayed too optimistically, thereby limiting the generalisability of findings beyond the scope of our research. At the same time, insights into early barriers and facilitators during the normative integration phase support the identification of areas for improvement towards functional integration and, as such, the scaling of the intervention.

### Conclusions

This study demonstrates that meaningful citizen involvement is a core determinant of successful implementation when serving socioeconomically vulnerable groups. Given the inherent complexity of such involvement, deliberate and methodologically informed approaches are a prerequisite for success. Beyond citizen involvement, findings highlight a critical tension between normative and functional integration: while shared values and professional enthusiasm represent a non-trivial accomplishment, for sustainable implementation they cannot substitute for formal governance structures and structural financing. Developing a learning health system represents a promising approach to generating evidence of effectiveness and fostering continuous improvement in support of functional integration.

## Additional File

The additional file for this article can be found as follows:

10.5334/ijic.10182.s1 Additional file 1. Supplementary Table 1: Complete list of identified barriers and facilitators.

## Data Availability

The datasets generated and analysed during the current study are not publicly available for privacy reasons.

## Abbreviations

CFIR – Consolidated Framework of Implementation Research

RMIC – Rainbow Model of Integrated Care

ERIC – Expert Recommendation for Implementing Change

nWMO – Research not falling under the Dutch Medical Research with Human Subjects Law

Q – Quote

## Author Information

Jonne G. ter Braake, Annefrans F.T.M. van Ede, Jessica Kiefte-de Jong, and Rimke C. Vos work in research for an academic hospital. Jonne G. ter Braake is doing her PhD on social determinants of cardiovascular care; Annefrans F.T.M. van Ede works as a postdoc on the implementation of Population Health Management; Jessica Kiefte-de Jong is a professor of Population Health; and Rimke C. Vos works as an associate professor of implementation of Population Health Management. Jonne G. ter Braake was present during the group consultation’s implementation team meetings in 2024 and Rimke C. Vos has been part of the implementation team since 2019. Tara Kwakkernaat and Maud Verhoeven were both students at the time of this study and were involved in data collection as part of her bachelor’s dissertation in Health Policy and Management and her master’s dissertation in Population Heath Management, respectively.

## References

[1] Beran M, Schram MT. Prediabetes in Nederland. Diabetes Fonds: Maastricht University; Maastricht UMC.

[2] Echouffo-Tcheugui JB, Selvin E. Pre-Diabetes and What It Means: The Epidemiological Evidence. Annual Review Public Health. 2021;42:59–77. DOI: 10.1146/annurev-publhealth-090419-102644 PMC8026645 33355476

[3] Ligthart S, van Herpt TTW, Leening MJG, Kavousi M, Hofman A, Stricker BHC, et al. Lifetime risk of developing impaired glucose metabolism and eventual progression from prediabetes to type 2 diabetes: a prospective cohort study. The Lancet Diabetes & Endocrinology. 2016;4(1):44–51. DOI: 10.1016/S2213-8587(15)00362-9 26575606

[4] Agardh E, Allebeck P, Hallqvist J, Moradi T, Sidorchuk A. Type 2 diabetes incidence and socio-economic position: a systematic review and meta-analysis. Int J Epidemiol. 2011;40:804–818. DOI: 10.1093/ije/dyr029 21335614

[5] Tabák AG, Herder C, Rathmann W, Brunner EJ, M K. Prediabetes: a high-risk state for diabetes development. Lancet. 2012;379(9833):2279–2290. DOI: 10.1016/S0140-6736(12)60283-9 22683128 PMC3891203

[6] American Diabetes A. 3. Prevention or Delay of Type 2 Diabetes: Standards of Medical Care in Diabetes—2021. Diabetes Care. 2020;44(Supplement_1):S34–S39. DOI: 10.2337/dc21-S003 33298414

[7] van Ommen B, Wopereis S, van Empelen P, van Keulen HM, Otten W, Kasteleyn M, et al. From Diabetes Care to Diabetes Cure-The Integration of Systems Biology, eHealth, and Behavioral Change. Frontiers in endocrinology. 2018;8(381). DOI: 10.3389/fendo.2017.00381 PMC5786854 29403436

[8] Chinn DJ, White M, Howel D, Harland JOE, Drinkwater CK. Factors associated with non-participation in a physical activity promotion trial. Public Health. 2006;120(4):309–319. DOI: 10.1016/j.puhe.2005.11.003 16473376

[9] Stange KC. The Problem of Fragmentation and the Need for Integrative Solutions. The Annals of Family Medicine. 2009;7(2):100. DOI: 10.1370/afm.971 19273863 PMC2653966

[10] Alley Dawn E, Asomugha Chisara N, Conway Patrick H, Sanghavi Darshak M. Accountable Health Communities — Addressing Social Needs through Medicare and Medicaid. New England Journal of Medicine. 2016;374(1):8–11. DOI: 10.1056/NEJMp1512532 26731305

[11] Béland F, Hollander MJ. Integrated models of care delivery for the frail elderly: international perspectives. Gaceta Sanitaria. 2011;25:138–146. DOI: 10.1016/j.gaceta.2011.09.003 22088903

[12] Cameron A, Lart R, Bostock L, Coomber C. Factors that promote and hinder joint and integrated working between health and social care services: a review of research literature. Health & Social Care in the Community. 2014;22(3):225–233. DOI: 10.1111/hsc.12057 23750908

[13] Nies H, Stekelenburg D, Minkman M, Huijsman R. A Decade of Lessons Learned from Integration Strategies in the Netherlands. International Journal of Integrated Care. 2021. DOI: 10.5334/ijic.5703 PMC8588908 34824564

[14] Wu J, Xue E, Huang S, Fu Y, Chen D, Shao J, et al. Facilitators and Barriers of Integrated Care for Older Adults with Multimorbidity: A Descriptive Qualitative Study. Clinical interventions in aging. 2023;18:1973–1983. DOI: 10.2147/CIA.S436294 38050622 PMC10693763

[15] Peer Y, Koren A. Facilitators and barriers for implementing the integrated behavioural health care model in the USA: An integrative review. International Journal of Mental Health Nursing. 2022;31(6):1300–1314. DOI: 10.1111/inm.13027 35637556

[16] Watt N, Sigfrid L, Legido-Quigley H, Hogarth S, Maimaris W, Otero-García L, et al. Health systems facilitators and barriers to the integration of HIV and chronic disease services: a systematic review. Health Policy and Planning. 2017;32(suppl_4):iv13–iv26. DOI: 10.1093/heapol/czw149 28666336 PMC5886067

[17] Engel M, Stoppelenburg A, van der Ark A, Bols FM, Bruggeman J, Janssens-van Vliet ECJ, et al. Development and implementation of a transmural palliative care consultation service: a multiple case study in the Netherlands. BMC Palliative Care. 2021;20(1):81. DOI: 10.1186/s12904-021-00767-6 34090394 PMC8180007

[18] Grootjans SJM, Stijnen MMN, Hesdahl-De Jong I, Kroese MEAL, Ruwaard D, Jansen MWJ. Implementation of an integrated community approach in deprived neighbourhoods: a theory-based process evaluation using the Consolidated Framework for Implementation Research (CFIR). Scandinavian Journal of Public Health. 2023:14034948231199804. DOI: 10.1177/14034948231199804 PMC11481404 37726916

[19] Zavrnik Č, Danhieux K, Monarres MH, Stojnić N, Lukančič MM, Martens M, et al. Scaling-up an integrated care for patients with non-communicable diseases: An analysis of healthcare barriers and facilitators in Slovenia and Belgium. Slovenian Journal of Public Health. 2021;60(3):158–166. DOI: 10.2478/sjph-2021-0023 34249162 PMC8256765

[20] Huber M, van Vliet M, Giezenberg M, Winkens B, Heerkens Y, Dagnelie PC, et al. Towards a ‘patient-centred’ operationalisation of the new dynamic concept of health: a mixed methods study. BMJ Open. 2016;6(1):e010091. DOI: 10.1136/bmjopen-2015-010091 PMC4716212 26758267

[21] Integraal Zorgakkoord. 2022.

[22] Ministerie van Volksgezondheid WeS. GALA Gezond en Actief Leven Akkoord. 2023.

[23] Skoglund G, Nilsson BB, Olsen CF, Bergland A, Hilde G. Facilitators and barriers for lifestyle change in people with prediabetes: a meta-synthesis of qualitative studies. BMC Public Health. 2022;22(1):553. DOI: 10.1186/s12889-022-12885-8 35313859 PMC8935766

[24] Proctor E, Silmere H, Raghavan R, Hovmand P, Aarons G, Bunger A, et al. Outcomes for Implementation Research: Conceptual Distinctions, Measurement Challenges, and Research Agenda. Administration and Policy in Mental Health and Mental Health Services Research. 2011;38(2):65–76. DOI: 10.1007/s10488-010-0319-7 20957426 PMC3068522

[25] Berwick DM, Nolan TW, Whittington J. The Triple Aim: Care, Health, And Cost. Health Affairs. 2008;27(3):759–769. DOI: 10.1377/hlthaff.27.3.759 18474969

[26] Leeftijd, achtergrond en sociaal-economische status [Available from: https://denhaag.incijfers.nl/mosaic/nl-nl/segregatiemonitor/2--leeftijd--achtergrond-en-sociaal-economische-status].

[27] Damschroder LJ, Reardon CM, Widerquist MAO, Lowery J. The updated Consolidated Framework for Implementation Research based on user feedback. Implementation Science. 2022;17(1):75. DOI: 10.1186/s13012-022-01245-0 36309746 PMC9617234

[28] Valentijn PP, Schepman SM, Opheij W, Bruijnzeels MA. Understanding integrated care: a comprehensive conceptual framework based on the integrative functions of primary care. International Journal of Integrated Care. 2013. DOI: 10.5334/ijic.886 PMC3653278 23687482

[29] Implementation TCf. Consolidated Framework for Implementation Research (CFIR). https://thecenterforimplementation.com/toolbox/cfir2025.

[30] Powell BJ, Waltz TJ, Chinman MJ, Damschroder LJ, Smith JL, Matthieu MM, et al. A refined compilation of implementation strategies: results from the Expert Recommendations for Implementing Change (ERIC) project. Implementation Science. 2015;10(1):21. DOI: 10.1186/s13012-015-0209-1 25889199 PMC4328074

[31] Hennink MM, Kaiser BN, Marconi VC. Code Saturation Versus Meaning Saturation: How Many Interviews Are Enough? Qualitative Health Research. 2016;27(4):591–608. DOI: 10.1177/1049732316665344 27670770 PMC9359070

[32] Rutakumwa R, Mugisha JO, Bernays S, Kabunga E, Tumwekwase G, Mbonye M, et al. Conducting in-depth interviews with and without voice recorders: a comparative analysis. Qualitative Research. 2019;20(5):565–581. DOI: 10.1177/1468794119884806 32903872 PMC7444018

[33] Ritchie J, Spencer L. Qualitative data analysis for applied policy research. In: Bryman A, Burgess RG, editors. Analysing qualitative data. London: Routledge; 1994. pp. 173–194. DOI: 10.4324/9780203413081_chapter_9

[34] Smith B, McGannon KR. Developing rigor in qualitative research: problems and opportunities within sport and exercise psychology. International Review of Sport and Exercise Psychology. 2018;11(1):101–121. DOI: 10.1080/1750984X.2017.1317357

[35] Strategy Design Consolidated Framework for Implementation Research [Available from: https://cfirguide.org/choosing-strategies/].

[36] Stronks K, Hoeymans N, Haverkamp B, den Hertog FRJ, van Bon-Martens MJH, Galenkamp H, et al. Do conceptualisations of health differ across social strata? A concept mapping study among lay people. BMJ Open. 2018;8(4):e020210. DOI: 10.1136/bmjopen-2017-020210 PMC5914775 29674369

[37] van der Vlegel-Brouwer W, Eelderink M, Bussemaker J. Participatory Action Research as a Driver for Health Promotion and Prevention: A Co-creation Process Between Professionals and Citizens in a Deprived Neighbourhood in the Hague. International Journal of Integrated Care. 2023. DOI: 10.5334/ijic.7560 PMC10691282 38047119

[38] van de Wetering S. Facilitating citizen participation in marginalised neighbourhoods: selective empowerment in between vulnerability and active citizenship. Local Government Studies. 2024;50(3):498–520. DOI: 10.1080/03003930.2023.2218801

[39] Krijgsheld M, Schmidt E, Levels E, Schuurmans M. Healthcare professionals as change agents: Factors influencing bottom-up, personal initiatives on appropriate care, a qualitative study in the Netherlands. Health Policy. 2024;147:105120. DOI: 10.1016/j.healthpol.2024.105120 38981279

[40] Berwick DM. Disseminating Innovations in Health Care. JAMA. 2003;289(15):1969–1975. DOI: 10.1001/jama.289.15.1969 12697800

[41] Holterman S, Lahr M, Wynia K, Hettinga M, Buskens E. Integrated Care for Older Adults: A Struggle for Sustained Implementation in Northern Netherlands. International Journal of Integrated Care. 2020. DOI: 10.5334/ijic.5434 PMC7366864 32742247

[42] Hilhorst P, Wijngaart Mvd. Samenwerking sociaal en medisch domein. Een verkenning. EMMA: EMMA; 2024.

[43] European Commission: Directorate-General for H, Food S. Blocks – Tools and methodologies to assess integrated care in Europe – Report by the Expert Group on Health Systems Performance Assessment: Publications Office; 2017.

[44] Reid RJ, Wodchis WP, Kuluski K, Lee-Foon NK, Lavis JN, Rosella LC, et al. Actioning the Learning Health System: An applied framework for integrating research into health systems. SSM – Health Systems. 2024;2:100010. DOI: 10.1016/j.ssmhs.2024.100010

