# Supplementary material for: Implementation Barriers and Facilitators of an Integrated Care Initiative Targeting Socioeconomically Vulnerable Groups
Source: Int J Integr Care. 2026 Aug 18;26(3):11. doi: 10.5334/ijic.10182 (PMC13496229; doi:10.5334/ijic.10182)
Supplement: Supplementary file 1 [file ijic-26-3-10182-s1.pdf]

Additional file 1

Supplementary Table 1: Complete list of identified barriers and facilitators

Domain	Construct	Clinical level	Professional level	Organisational level	System level
Innovation	Innovation source			+ Conceived based on personal experience of the initiator	
	Innovation evidence-base			+ The intervention is composed of evidence-based components	
	Innovation relative advantage	+ Authority of general practitioner<br>+ Presence of different disciplines perceived as interesting<br>+ The measurements are appreciated and seen as the primary reason for going to the group consultation	+ Peer support<br>+ Authority of general practitioner<br>+ Multidisciplinary design contributes to recognisability and diversity of professionals involved<br>+ Concrete measurements raise participants' awareness and provide immediate guidance for next steps from different professionals<br>+ Low-threshold nature for participation<br>- Intervention potentially suitable for a narrow group	+ More time to talk about participants' needs<br>+ Instant guidance for follow-up steps from different professionals where a warm handoff is achieved	+ Equality of professionals and residents, no white coat syndrome
	Innovation adaptability	+ Theme can be adapted to health care needs of target group	+ Theme can be adapted to health care needs of target group	+ Theme can be adapted to health care needs of target group<br>+ Location and setting can be adapted based on needs	
	Innovation trialability				
	Innovation complexity			- Themes of health needs do not always align with funding streams<br>- Setting makes participants	- Themes of health needs do not always align with funding streams

				hesitant to share personal data	
	Innovation design	≈ Key to creating a sense of trust during the group consultation<br>≈ Participating in the group consultation should be low threshold<br>- Dutch language and use of jargon<br>- The examples do not sufficiently focus on the cultural background of participants<br>- Participants receive too little guidance in moving to follow-up initiatives	- Dutch language and use of jargon<br>- The examples do not sufficiently focus on the cultural background of participants<br>- Insufficient connection to follow-up initiatives (both the flow of participants and feedback from professionals)<br>- Ideal group size ambiguous<br>- 'Group consultation' is an ambiguous name<br>- Participation is complicated for employed people because group consultations and follow-up initiatives are only offered during office hours	- Dutch language and use of jargon<br>- Insufficient connection to follow-up initiatives (both the flow of participants and feedback from professionals)<br>- Ideal group size ambiguous<br>- A blueprint of the group consultations is lacking	- Ideal number of professionals relative to group size unclear<br>- Business operations are missing. There is currently no concept to embed, there must first be a clearly delineated definition of 'the group consultation', for which a business model can be developed. Then this needs to be embedded in formal structures
	Innovation cost			- The group consultation is expensive	- The group consultation is expensive
Outer setting	Critical incidents				
	Local attitudes	+ The societal view on health<br>- It is difficult to share private matters in a group; from the cultural background of many residents, these matters must remain private<br>- Many residents find it important to have separate groups for men and for women.	+ The societal view on health<br>- Residents are looking for a short-term solution when they develop health issues	+ The societal view on health	+ The societal view on health
	Local conditions	≈ People are part of an informal network which influences who they do and do not trust	+ Unhealthy population<br>+ There is a gap between supply and demand for social support	+ Unhealthy population<br>+ Increased need for welfare initiatives<br>- Fragmentation of funding	+ There is a gap between supply and demand for social support<br>+ There is formal attention to

		- Large social distance between residents and professionals reduces trust<br>- Residents have little confidence in the system<br>- Residents face many different problems		- High workload among professionals	the importance of prevention at the national level<br>- Lack of formal responsibility for health<br>- Movable political climate both at the national and municipal level
	Partnerships and connections		≈ Collaborational networks of professionals	≈ Collaborational networks of professionals	≈ Collaborational networks of payers<br>+ A cross-domain payment title
	Policies and laws			- Legal demarcation of the medical and social domains for funding	- Legal demarcation of the medical and social domains for funding
	Financing			- Due to lack of formal responsibility, parties point to each other for funding	+ Availability of temporary budgets<br>- Structural budgets are not available
	External pressure		+ Media coverage of health issues		+ Attention to the importance of interdisciplinary work<br>- Lack of financial incentive for prevention
Inner setting	Structural characteristics		- Aligning different work schedules<br>- Sick leave of personnel	- High working pressure	- Involved professionals use different ICT tools, thereby challenging communication.
	Relational connections		≈ Formal and informal networks between professionals in the neighbourhood	≈ Formal and informal networks between professionals in the neighbourhood	
	Communications		≈ A formal and informal network is the prerequisite for low-threshold communication	≈ A formal and informal network is the prerequisite for low-threshold communication	
	Culture		≈ Willingness to change<br>+ Egalitarian relationships between professionals	≈ Trust and respect	
	Tension for change		+ Many relapses frustrate professionals		

	Compatibility				
	Relative priority				
	Incentive systems				
	Mission alignment			+ Strong support for the concept of the group consultations	
	Available resources	≈ Important that involved residents are paid for work they provide for the initiative<br>- Citizens have to decide between joining the group consultation and other responsibilities	≈ Low-threshold location for the target group<br>- Money	≈ Low-threshold location for the target group<br>+ Inkind contributions from welfare organisations<br>- Money<br>- Temporary funding brings with it obligations, e.g. conducting research	- Because temporary funding is available, the need to arrange sustainable funding is lacking<br>- In-kind funding may cease<br>- Temporary funding brings with it obligations, e.g. conducting research
	Access to knowledge and information				
Individuals	High-level leaders			- Dependence on intrinsic motivation of professionals in influential roles	- Dependence on intrinsic motivation of professionals in influential roles<br>- Reluctance to invest in temporary initiatives<br>- Health insurers (and other payers) have different views on what is needed
	Mid-level leaders			- Professionals have to work against the mainstream current	- Varying views from professionals on the importance of prevention
	Opinion leaders				
	Implementation facilitators				
	Implementation leads	+ Enthusiasm about the initiator		- Roles ambiguous	+ Enthusiasm of initiator motivates stakeholders<br>- Group consultation depends on initiator, embedding is

					lacking
	Implementation team members	+ Experts by experience perceive contributing to the group consultations as a great way to step outside their current network<br>+ Experts by experience are close to the residents and therefore well equipped to motivate residents	≈ Importance of (a few) enthusiastic people leading the initiative	+ Enthusiasm among professionals about group consultations	
	Other implementation support			+ Professional background of volunteers promotes implementation<br>- Professional expertise of volunteers is not rewarded proportionately	
	Innovation deliverers		+ Enthusiasm among professionals about group consultations	+ Enthusiasm among professionals about group consultations	
	Innovation recipients	- There is varying motivation to exercise, for example walking<br>- Little motivation to change own situation	- The target group sets other priorities over exercise and health, especially if they are not yet 'sick'<br>- Employed people are not available during office hours		
Process	Teaming				≈ Actively involve the necessary (paying) parties in the implementation process at an early stage
	Assessing needs	- The group consultations are still too much from the professionals' perspective, important to adapt it to the needs of participants	- Residents are not sufficiently involved in the design of group consultations	- Residents are not sufficiently involved in the design of group consultations	
	Assessing context				

	Planning	- Need for an annual planning with meetings on various themes		- There is a lack of embedding and a long-term vision<br>- A blueprint and clear roles are lacking	- Not enough thought is given to structured scaling
	Tailoring strategies				
	Engaging		- Group consultation lacks awareness among professionals and residents<br>- Inflow of residents to group consultations is low<br>- Lack of feedback to professionals reduces engagement	- Inflow of residents to group consultations is low	- Inflow of residents to group consultations is low
	Doing		≈ Scheduling group consultations ahead<br>+ Continuous adaptation of the intervention based on experience	+ The intervention just started and the team gradually learns from the process<br>- There is a lot of talk but little action	- There is a lot of talk but little action
	Reflecting and evaluating		- There is no structured evaluation on effectiveness and process	≈ Effectiveness of prevention is difficult to quantify<br>- There is no structured evaluation on effectiveness and process	≈ Effectiveness of prevention is difficult to quantify<br>- Insufficient effectiveness evaluation to generate supporting evidence
	Adapting			+ Setting of the intervention can be adapted to the target group	
